# The *Pseudomonas aeruginosa* Type VI secretion system toxin Tse8 evolved from a novel *N*-carbamoylputrescine amidohydrolase

**DOI:** 10.1042/BCJ20253210

**Published:** 2025-07-22

**Authors:** Bin Li, Hamid R. Baniasadi, Margaret A. Phillips, Anthony J. Michael

**Affiliations:** 1 Department of Biochemistry, University of Texas Southwestern Medical Center, Dallas, Texas TX 75390, U.S.A.

**Keywords:** agmatine deiminase, convergent evolution, *N*-carbamoylputrescine amidohydrolase, polyamine, *Pseudomonas aeruginosa*, Type VI secretion system toxin

## Abstract

The polyamine putrescine is synthesized primarily from L-arginine via agmatine in bacteria. There are currently three known routes from agmatine to putrescine, including direct conversion by agmatinase. The other two routes use agmatine deiminase to produce *N*-carbamoylputrescine from agmatine, then one of two nonhomologous enzymes, putrescine transcarbamylase or *N*-carbamoylputrescine amidohydrolase (NCPAH), converts *N*-carbamoylputrescine to putrescine. Here, we functionally identify enzymes from phylogenetically distant bacteria, the ɣ-proteobacterium *Shewanella oneidensis*, and the actinomycetota species *Microterricola gilva*, that are novel alternative, nonhomologous, noncanonical NCPAHs that we term AguY, which have emerged by convergent evolution. Kinetic analysis indicates that the AguY enzymes are as efficient as the canonical NCPAH from *Pseudomonas aeruginosa* in converting *N*-carbamoylputrescine to putrescine. Genomic evidence suggests that the AguY enzymes may participate in putrescine biosynthetic or agmatine catabolic pathways and are occasionally encoded in genomes that also encode agmatinase. We show that the Type VI secretion system toxin Tse8 from *P. aeruginosa* has evolved from AguY. It is formally possible that AguY evolved directly or indirectly from the ancient glutamine amidohydrolase GatA, a component of the transamidosome, an RNA/protein complex required for the production of glutamine-charged tRNA. Our study provides a further example of the prevalence of convergent evolution and horizontal gene transfer in polyamine biosynthesis, suggesting pervasive selective pressure to evolve polyamine metabolism in bacteria.

## Introduction

Convergent biochemical evolution can be defined as the independent origin of different biochemical functional modules that convert the same substrate into the same product. It can operate at the level of single enzymes or whole metabolic pathways. For individual enzymes, the most distinct form of convergent evolution is nonhomologous proteins from different structural folds that perform the same biochemical function, known as nonhomologous isofunctional enzymes [1–3]. Recently, three paradigms for convergent evolution of enzymes (isozymes) have been proposed [3]. The first paradigm includes enzymes that have two or more catalytic residues aligned in 3D, with similarities in reaction mechanism. The second includes enzymes with similar reaction mechanisms but with no aligned catalytic residues. In the third paradigm, only the overall reaction is similar, with no reaction mechanism or active site similarity.

**Figure 1:**
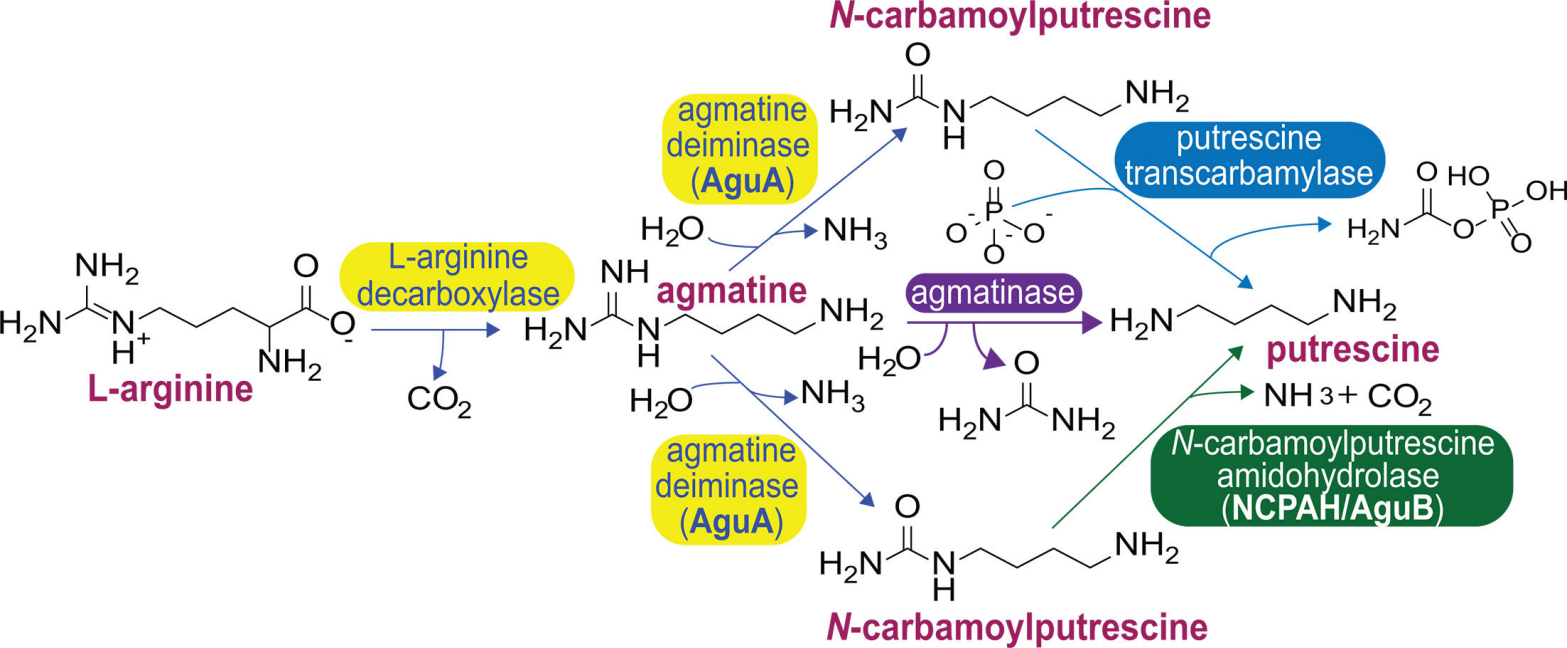
Convergent pathways from agmatine to putrescine.

Polyamines are small flexible organic polycations that are found throughout the tree of life [4]. The most common polyamines are the diamine putrescine and the triamine spermidine. It is likely that spermidine synthase, and therefore spermidine, was present in the Last Universal Common Ancestor of all life [5]. In many phylogenetically diverse bacteria, the diamine putrescine is synthesized indirectly from L-arginine [6]. The first step of this pathway is decarboxylation of L-arginine by arginine decarboxylase (ADC) to produce agmatine (Figure 1). There are at least five distinct examples of convergent evolution of ADC, each from a different protein fold [7,8]. Two convergent forms of ADC are dependent on pyridoxal 5′-phosphate (PLP) as a cofactor, exemplified by the *Escherichia coli* biosynthetic ADC (SpeA) [9] and *Bacillus subtilis* SpeA [10,11]. The other three convergent forms are dependent on an internally generated pyruvoyl group as cofactor, exemplified by the *Methanocaldococcus jannachii* ADC [12], *Sulfolobus solfataricus* ADC [13], and *Aspergillus oryzae* ADC [8].

The conversion of agmatine to putrescine has also been subject to convergent evolution. The most biochemically characterized pathway is the direct ureohydrolysis of agmatine to putrescine by the manganese-dependent enzyme agmatine ureohydrolase (agmatinase, SpeB) (Figure 1). Agmatinase (EC 3.5.3.11) is a binuclear manganese metalloenzyme [14–17] that was first isolated from *E. coli* [18–20], and then identified in *B. subtilis* [10]. In the agmatinase reaction, water is used to release urea from the hydrolysis of L-arginine to produce putrescine. The agmatinase pathway is known primarily as a biosynthetic step, but it is also known to be involved in catabolism of L-arginine and agmatine. A homologous enzyme, arginase (EC 3.5.3.1), converts L-arginine to L-ornithine and urea [21]. In contrast to the direct ureohydrolysis of agmatine to putrescine and urea, two other pathways are known that first hydrolyze agmatine to *N*-carbamoylputrescine and ammonia, and then convert *N*-carbamoylputrescine to putrescine [22]. Both pathways (Figure 1) use the same agmatine deiminase (AguA) enzyme (EC 3.5.3.12) to produce *N*-carbamoylputrescine [23,24]. One pathway is known as the agmatine deiminase system, which converts *N*-carbamoylputrescine and phosphate to putrescine and carbamoyl phosphate using the enzyme putrescine transcarbamylase (EC 2.1.3.6), and is a catabolic pathway that generates ATP from the catabolism of agmatine [25,26]. Putrescine transcarbamylase is homologous to ornithine transcarbamylase (EC 2.1.3.3), a component of the arginine deiminase system [27,28].

A second pathway (Figure 1) from agmatine and *N*-carbamoylputrescine to putrescine was first identified in *Pseudomonas aeruginosa* and consists of AguA and *N*-carbamoylputrescine amidohydrolase (NCPAH/AguB) [29–33]. AguB (EC 3.5.1.53), a nitrilase-like enzyme, converts *N*-carbamoylputrescine to putrescine and ammonia, and the AguA–AguB pathway is primarily anabolic in bacteria and plants but is also used for agmatine catabolism in bacteria. The putrescine transcarbamylase and AguB protein structures evolved from different protein folds and are not homologous [34,35]. They represent convergently evolved alternative enzymes for the conversion of *N*-carbamoylputrescine to putrescine but which differ in their reaction mechanisms. We have now identified a further example of convergent evolution in the pathway from *N*-carbamoylputrescine to putrescine, an alternative form of NCPAH/AguB, that is not homologous to the canonical AguB but which also produces putrescine and ammonia from *N*-carbamoylputrescine.

## Results

### Genomic evidence for a novel noncanonical *N*-carbamoylputrescine amidohydrolase

**Figure 2:**
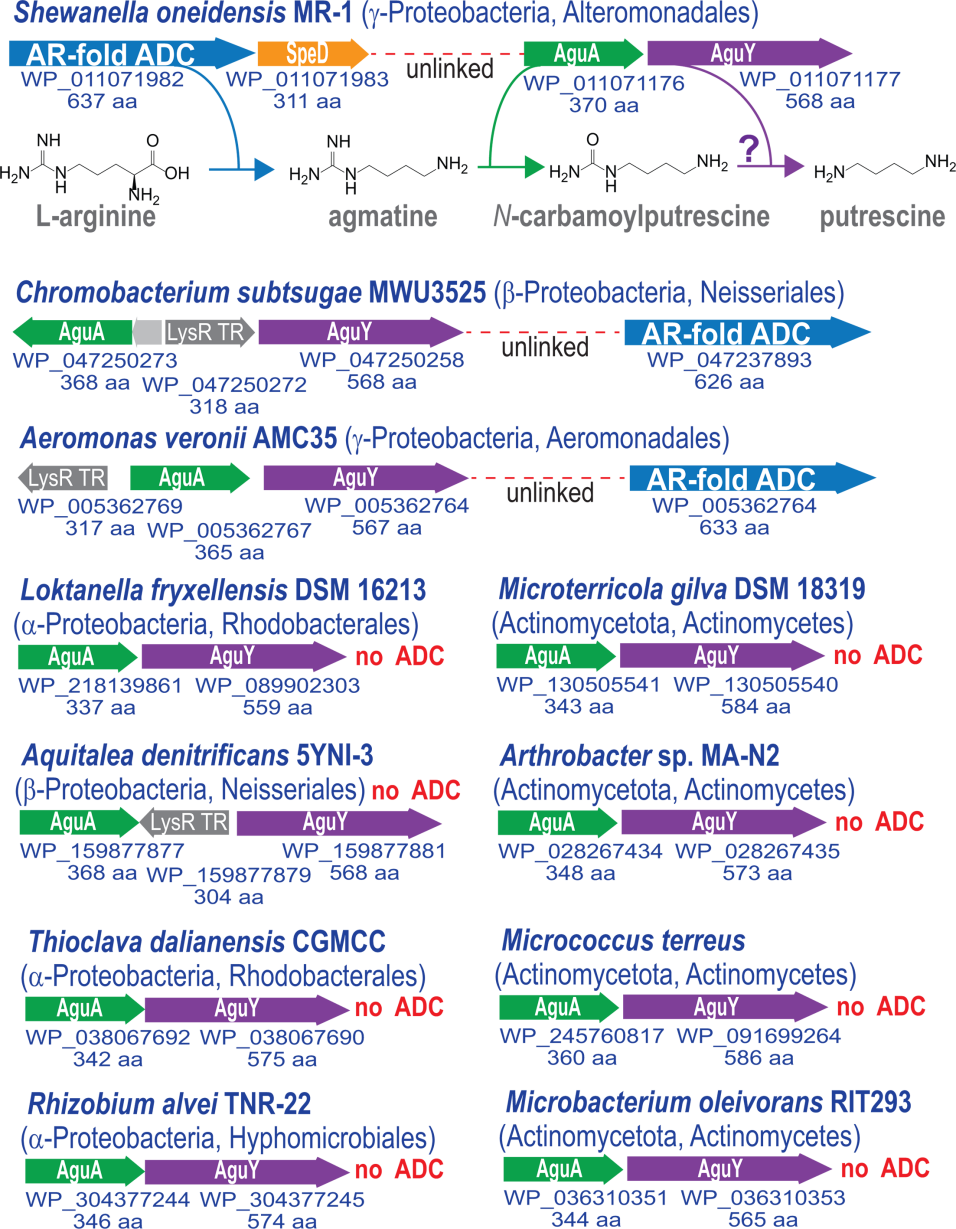
The presence of a potential alternative *N*-carbamoylputrescine amidohydrolase ORF in bacterial genomes. AR-fold ADC, alanine racemase-fold arginine decarboxylase; AguA, agmatine deiminase; AguY, alternative *N*-carbamoylputrescine amidohydrolase; LysR TR, LysR-type transcription factor; SpeD, *S*-adenosylmethionine decarboxylase.

The γ-proteobacterium *Shewanella oneidensis* accumulates putrescine as its only polyamine [36]. We noticed that *S. oneidensis* MR-1 encodes a typical alanine racemase fold ADC, similar to the *E. coli* and *Campylobacter jejuni* ADCs [6,37], that could produce agmatine from L-arginine (Figure 1). It also encodes an AguA homolog to convert agmatine to *N*-carbamoylputrescine (Figure 1). However, the *S. oneidensis* genome does not encode a canonical NCPAH/AguB homolog nor a putrescine transcarbamylase homolog. Immediately downstream of the *aguA* gene is an open reading frame (ORF) encoding an amidase homolog (WP_011071177; 568 aa) that is not homologous to the canonical AguB and which is approximately twice the size (Figure 2). We found the amidase ORF adjacent to an *aguA* ORF in diverse bacteria in the α-, β-, and γ-Proteobacteria classes of the Pseudomonadota phylum, and in the phylogenetically distant Actinomycetota phylum. The consistent genomic juxtaposition of the AguA and amidase-encoding ORFs suggests that the amidase homolog may encode NCPAH activity and potentially represent a novel nonhomologous alternative NCPAH/AguB that we designate here as AguY. In some bacterial genomes containing ORFs encoding AguA and AguY, an ADC homolog is present, but in others, it is absent. The absence of ADC from a genome may indicate that in some bacteria, the AguA–AguY pathway functions in the catabolism of exogenously acquired agmatine.

### Biochemical evidence for a noncanonical *N*-carbamoylputrescine amidohydrolase

To determine whether the *S. oneidensis* AguY exhibits NCPAH activity, we coexpressed the *P. aeruginosa aguA* gene with the *S. oneidensis aguY* gene in an agmatinase gene deletion strain of *E. coli* BL21 (BL21*speB*). Absence of the agmatinase gene in the BL21*speB* strain results in an accumulation of agmatine that cannot be converted to putrescine (Li 24), and *E. coli* does not encode an AguA homolog. The *Enterococcus faecalis aguA* gene, working in concert with the putrescine transcarbamylase gene, is part of the agmatine deiminase system. Expression of the *P. aeruginosa or E. faecalis aguA* genes alone in BL21*speB* would be expected to cause an accumulation of *N*-carbamoylputrescine that cannot then be converted to putrescine. To detect *N*-carbamoylputrescine by LC-MS/MS, we decided to avoid benzoylation of cell extracts because we have found that benzoylation causes the complete hydrolysis of pure *N*-carbamoylputrescine. After the expression of either the *P. aeruginosa or E. faecalis aguA* genes alone from pETDuet-1 in BL21*speB*, a substantial accumulation of *N*-carbamoylputrescine was detected (Table 1). Although the expression of different genes expressed from pETDuet-1 cannot be directly compared due to potential differences in resultant steady-state protein levels, the approximate level of *N*-carbamoylputrescine accumulated was similar for each *aguA* gene. Coexpression of the *P. aeruginosa aguA* gene with the *P. aeruginosa* canonical *aguB* gene would be expected to decrease the level of *N*-carbamoylputrescine by conversion to putrescine. Indeed, coexpression of the *P. aeruginosa aguA* and *aguB* genes reduced *N*-carbamoylputrescine levels approximately ten-fold (Table 1). Coexpression of the *P. aeruginosa aguA* gene with the *S. oneidensis aguY* gene caused an approximately 200-fold decrease in *N*-carbamoylputrescine accumulation (Table 1). These data suggest that the *S. oneidensis aguY* gene encodes *N*-carbamoylputrescine amidohydrolase activity.

**Table 1:** LC-MS/MS analysis of underivatized *N*-carbamoyl putrescine content

*E. coli* BL21*speB* strain	AUP (NCP)
pETDuet-1 empty	2.52×10^4^
*P. aeruginosa* AguA	3.04×10^7^
*E. faecalis* AguA	2.30×10^7^
pETDuet-1 empty + pACYCDuet-1 empty	2.59×10^4^
*P. aeruginosa* AguA + *P. aeruginosa* AguB	2.30×10^6^
*P. aeruginosa* AIH + *S. oneidensis* AguY	1.27×10^5^

Agmatine deiminases (AguA) expressed from pETDuet-1, canonical AguB or AguY *N*-carbamoylputrescine amidohydrolases expressed from pACYCDuet-1. All strains were grown and analyzed together. Shown are area under the peak (AUP) values for underivatized *N*-carbamoylputrescine (NCP). AUP quantified with the 115.3 m/z daughter ion of the 132.1 m/z parental ion.

**Figure 3:**
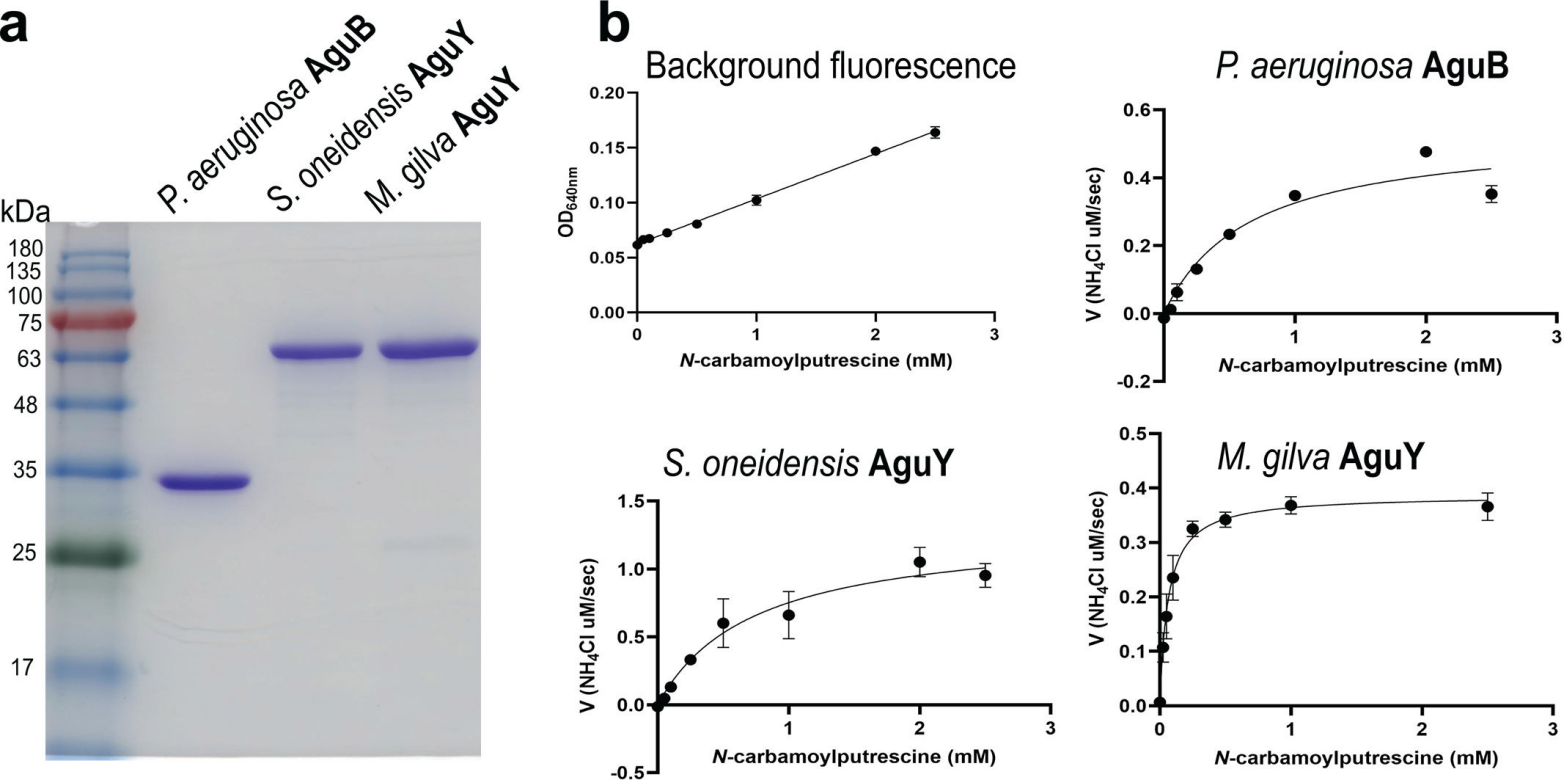
Kinetic analysis of purified recombinant canonical and alternative *N*-carbamoylputrescine amidohydrolases. (**a**) Purified recombinant canonical AguB, and AguY *N*-carbamoylputrescine amidohydrolases. (**b**) Mean reaction rates with varying concentrations of *N*-carbamoylputrescine, from triplicate reactions. Error bars show ± S.D.

Previously, a gene deletion spanning the *S. oneidensis aguA* and *aguY* genes (Figure 2) was found to result in depletion of the putrescine-containing siderophore putrebactin [38], which would be consistent with the *aguY* gene encoding an alternative NCPAH enzyme for putrescine production. However, in principle, the same result could be obtained with deletion of only the *aguA* gene. Furthermore, the *E. coli* BL21*speB* strain contains putrescine produced by the constitutive L-ornithine decarboxylase that confounds detection of putrescine synthesized from agmatine produced by coexpression of *aguA,* and *aguB* or the *S. oneidensis aguY* gene. To circumvent this problem, we purified recombinant *P. aeruginosa* AguB, the *S. oneidensis* AguY, and an AguY homolog from the Actinomycetota species *Microterricola gilva* (Figure 3a). The lengths and theoretical masses of the encoded proteins are as follows: *P. aeruginosa* AguB, 292 a.a. (32,780 Da); *S. oneidensis* AguY, 568 a.a. (60,996 Da); *M. gilva* AguY 584 a.a. (61,738 Da). Each protein was incubated with *N*-carbamoylputrescine, and LC-MS/MS was used to detect underivatized putrescine (Table 2). A background level of putrescine from the *N*-carbamoylputrescine was detected in the absence of proteins. Incubation with increasing amounts of protein increased the level of putrescine detected after 60 min of incubation. Similar levels of putrescine were produced by each protein at 50 nM. These data indicate that the *S. oneidensis* and *M. gilva* AguY proteins are catalytically active and exhibit NCPAH activity *in vitro* and, therefore, represent a new, independently evolved, alternative form of NCPAH enzyme.

**Table 2:** LC-MS/MS detection of *in vitro* putrescine production from *N*-carbamoylputrescine by purified recombinant amidohydrolases

	AUP			
	**Group 1**		Group 2	
Enzyme nM	*Pa*AguB	*So*AguY	*So*AguY	*Mg*AguY
0	3.13×10^4^	ND	6.91×10^4^	7.18×10^4^
10 (boiled)	2.42×10^4^	ND	4.81×10^4^	4.73×10^4^
10	1.89×10^6^	1.70×10^5^	6.72×10^5^	2.21×10^5^
25	2.95×10^6^	4.09×10^5^	1.97×10^6^	8.86×10^5^
50	2.80×10^6^	1.34×10^6^	3.01×10^6^	1.87×10^6^
100	NP	NP	4.47×10^6^	2.83×10^6^

Area under the Peak (AUP) values for underivatized putrescine detected by LC-MS/MS. Group 1 and Group 2 are independent experiments. *Pa* AguB, *P. aeruginosa N*-carbamoylputrescine amidohydrolase; *So* AguY, *S. oneidensis* alternative *N*-carbamoylputrescine amidohydrolase; *Mg* AguY, *M. gilva* AguY. ND, not detected; NP, not perfomed. Enzyme reactions were carried out at 40ºC for 60 min.

ND, not detected; NP, not performed.

### Kinetic analysis of noncanonical *N*-carbamoylputrescine amidohydrolases

To biochemically characterize and validate the catalytic activity of the potential alternative NCPAH proteins, we performed *in vitro* kinetic analysis of the *P. aeruginosa* AguB, and the *S. oneidensis* and *M. gilva* AguY proteins (Table 3). Catalytic activity was monitored by measuring released ammonia after *N*-carbamoylputrescine hydrolysis (Figure 3b). The *P. aeruginosa* AguB and *S. oneidensis* AguY proteins exhibited similar substrate affinity (*K*_m_) for *N*-carbamoylputrescine, but substrate turnover (*k*_cat_) was 2.5-fold faster for the *S. oneidensis* AguY protein (Table 3). In contrast, the *M. gilva* AguY exhibited a *K*_m_ for *N*-carbamoylputrescine that was ten-fold lower than for the other two proteins, but its *k*_cat_ was lower. The catalytic efficiency (*k*_cat_/*K*_m_) of the *M. gilva* AguY for converting *N*-carbamoylputrescine to putrescine was 7.8-fold higher than that of the *P. aeruginosa* AguB enzyme, and 3.6-fold higher than the *S. oneidensis* enzyme (Table 3). These data confirm that the *S. oneidensis* and *M. gilva* AguY proteins are alternative nonhomologous forms of NCPAH/AguB.

**Table 3:** Kinetic parameters determined from AguB and AguY recombinant proteins

Species	Protein	*K*_m_ (mM)	*k*_cat_ (s^-1^)	*k*_cat_/*K*_m_ (M^-1^s^-1^)
*Pseudomonas aeruginosa* AguB	NP_248984	0.66±0.11	54.1±3.0	82,500±8800
*Shewanella oneidensis* AguY	NP_716519	0.74±0.13	130.0±4.5	179,400±28,600
*Microterricola gilva* AguY	WP_130505540	0.07±0.3	39.0±1.5	641,800±250,900

All assays were performed with different concentrations of *N*-carbamoylputrescine, in triplicate at 40°C (± S.D.), with 10 nM enzyme at pH 7.0 for 30 min.

### Canonical and noncanonical *N*-carbamoylputrescine amidohydrolases, and agmatinases, in *Shewanella* species

**Figure 4:**
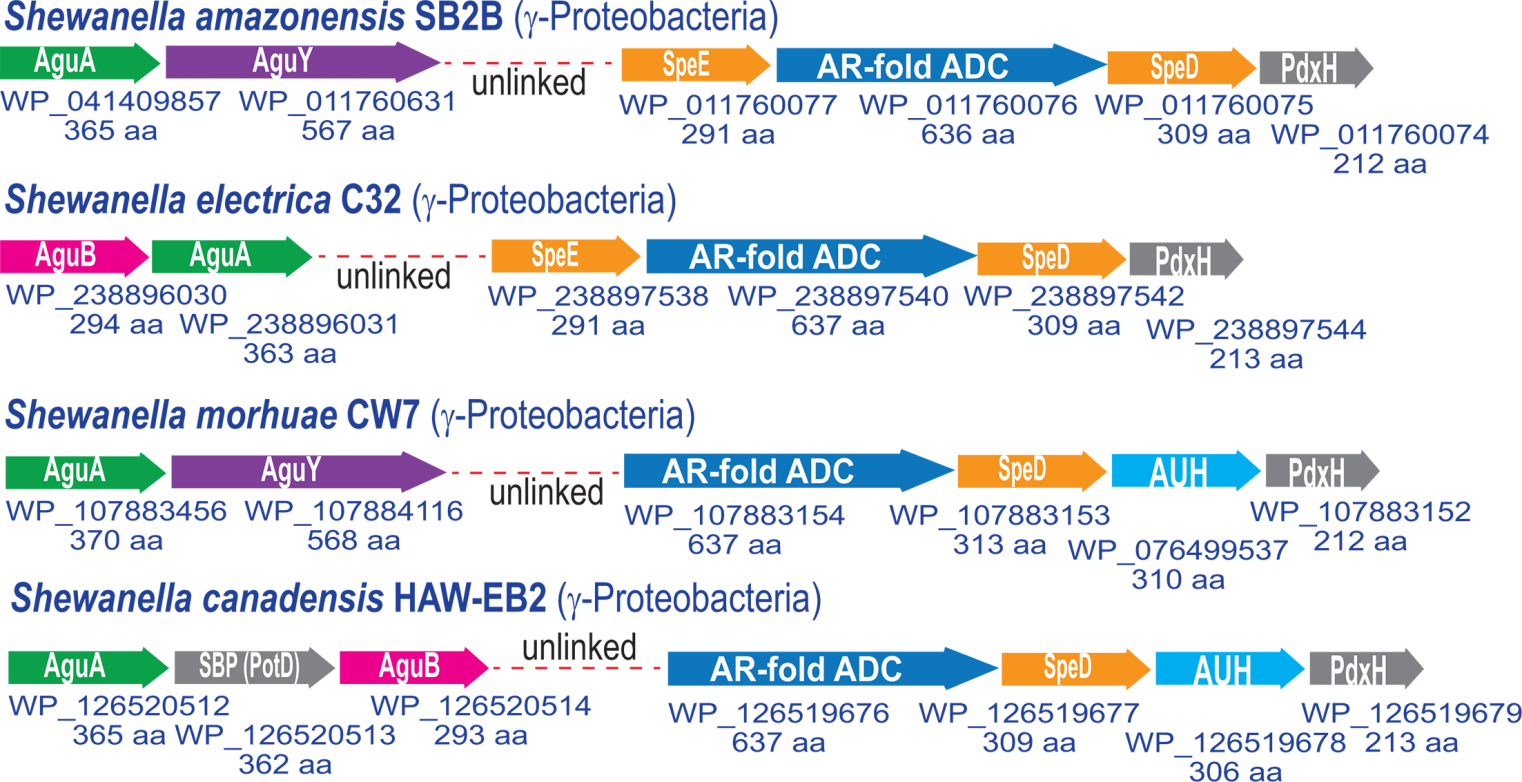
Gene clusters from different *Shewanella* species. AR-fold ADC, alanine racemase-fold arginine decarboxylase; AguA, agmatine deiminase; AguB, canonical *N*-carbamoylputrescine amidohydrolase; AguY, alternative *N*-carbamoylputrescine amidohydrolase; AUH, agmatine ureohydrolase/agmatinase (SpeB); PdxH, pyridoxine/pyridoxamine 5’-phosphate oxidase; SBP (PotD), polyamine substrate binding protein of ABC transporter; SpeD, *S*-adenosylmethionine decarboxylase, SpeE, aminopropyltransferase.

Phylogenetically diverse bacterial genomes contain ORFs encoding homologs of AguA adjacent to AguY-encoding ORFs. However, some of these genomes do not encode an ADC from any known protein fold (Figure 2). This suggests that in some bacterial species, the role of the AguA–AguY pathway is catabolism of exogenously derived agmatine. Once agmatine is converted to putrescine via *N*-carbamoylputrescine, it may be further catabolized to succinate and enter the TCA cycle [39]. In contrast, the AguY is likely to be required for biosynthesis of putrescine from L-arginine in species such as *S. oneidesis*, since this species encodes an ADC and AguA but does not encode a canonical NCPAH/AguB or an agmatinase. Within the *Shewanella* genus, some species encode the canonical NCPAH/AguB, e.g. *S. electrica*, whereas others encode AguY, e.g. *S. amazonensis* (Figure 4). A further layer of complexity is seen in other *Shewanella* species, where AguA and the canonical NCPAH/AguB or AguY are encoded by the same genomes that also encode an agmatinase homolog, e.g. *S. morhuae* with AguY, and *S. canadensis* with the canonical NCPAH/AguB (Figure 4). In these cases, it is likely that the agmatinase is involved in putrescine biosynthesis from L-arginine due to the presence of an ADC-encoding ORF in the same gene cluster as the agmatinase. This suggests that the canonical NCPAH/AguB or AguY encoded in these same genomes are involved in agmatine catabolism, consistent with an ABC transporter polyamine substrate binding protein homolog ORF being found between the AguA- and canonical NCPAH/AguB-encoding genes in *S. canadensis* (Figure 4). It is notable that the ADC-encoding gene clusters in *S. amazonensi*s, *S. electrica*, *S. morhuae,* and *S. canadensis* also encode a homolog of PdxH, i.e. pyridoxine/pyridoxamine 5′-phosphate oxidase, the last step in PLP biosynthesis. PLP is the cofactor of the ADC homologs encoded in these *Shewanella* species, and the presence of the *pdxH* gene suggests that PLP may be limiting for ADC activity.

### Type VI secretion system toxin Tse8 evolved from a noncanonical *N*-carbamoylputrescine amidohydrolase

**Figure 5:**
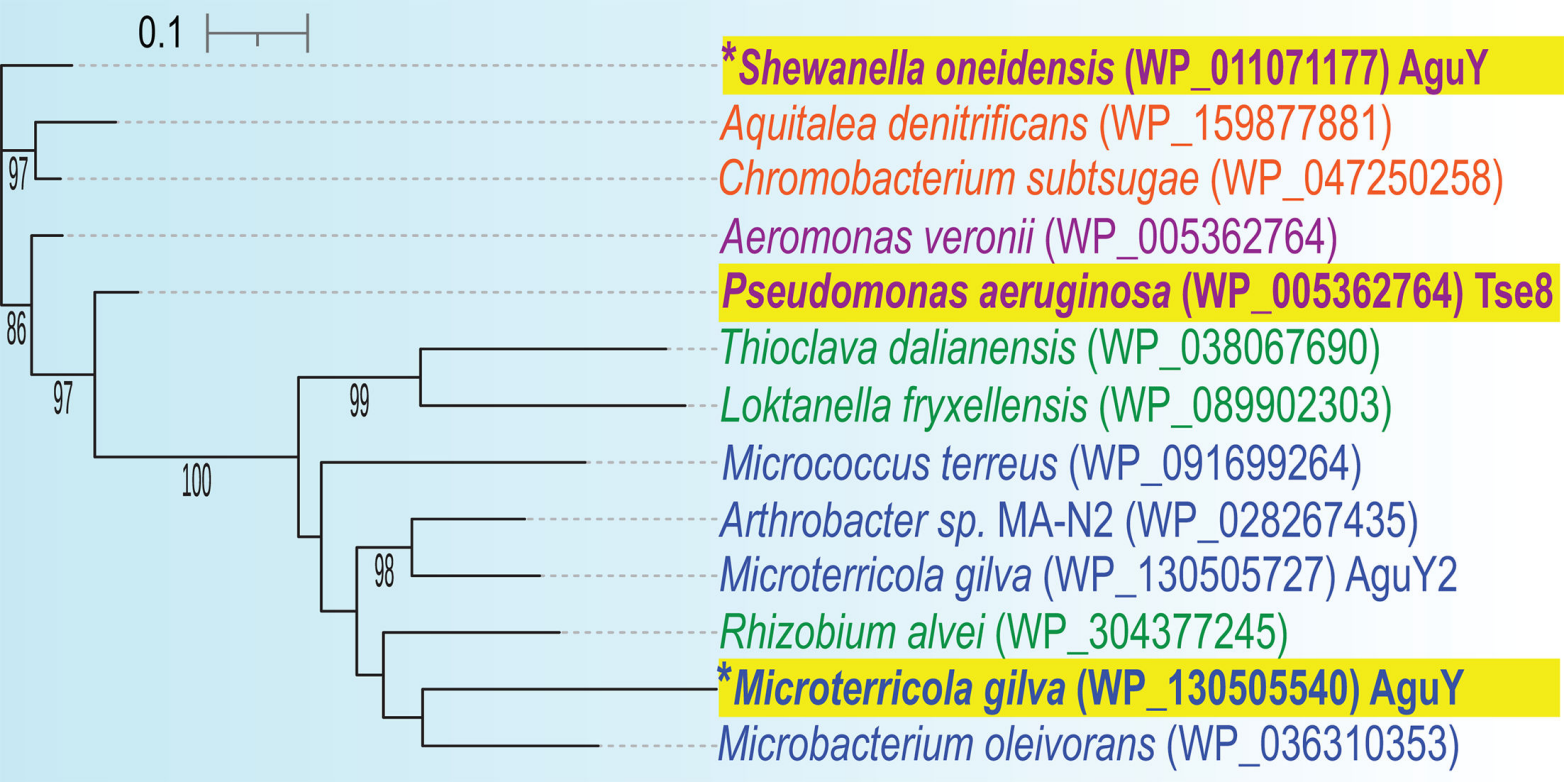
Maximum likelihood phylogenetic tree of alternative *N*-carbamoylputrescine amidohydrolase homologs. Actinomycetota phylum, blue; α-Proteobacteria class, green; β-Proteobacteria class, red; γ-Proteobacteria class, purple. Asterisks indicate biochemically validated proteins. All AguY homologs except Tse8 are found adjacent to AguA ORFs (Figure 2). Numerical support values above 80% from 1000 ultrafast bootstrap analyses are displayed. The scale bar represents the average number of amino acid substitutions per site.

We noticed that an AguY homolog (WP_005362764; 569 a.a.) is encoded by *P. aeruginosa* PAO1. The encoding gene is not adjacent to an *aguA* ORF, and the *P. aeruginosa* AguY homolog was previously identified as a Type VI secretion system toxin known as Tse8 [40]. Whereas the *S. oneidensis* and *M. gilva* biochemically validated NCPAH/AguY proteins are 65% identical (367/564 a.a.), the *S. oneidensis* AguY and *P. aeruginosa* Tse8 proteins are 85% identical (479/566 a.a.), and the Tse8 and *M. gilva* AguY proteins are 69% identical (394/568 a.a.). A maximum likelihood (ML) phylogenetic tree was constructed for the AguY homologs depicted in Figure 2 that are adjacent to AguA-encoding ORFs. A second paralog of AguY encoded in the *M. gilva* genome was also included. The *P. aeruginosa* Tse8 protein is found nested among AguY homologs, between the *S. oneidensis* and *M. gilva* AguY proteins (Figure 5). This ML phylogenetic tree reveals that the Type VI secretion system toxin Tse8 evolved from an AguY protein. The canonical AguA- and AguB-encoding genes were first identified in *P. aeruginosa* PAO1 as genes required for growth on agmatine as sole carbon and nitrogen source [29]. It is unlikely that Tse8 retains a function in agmatine utilization or putrescine biosynthesis as it is secreted from the cell [40]. The 3D structure of Tse8 shows that it is very similar to the Glutamyl-tRNA^Gln^ amidotransferase subunit A (GatA) from *Staphylococcus aureus* that converts glutamine to glutamate and ammonia [41]; however, Tse8 does not act on glutamine [40]. There is only 22% (125/560) amino acid identity between the *P. aeruginosa* Tse8 and GatA (WP_003112871; 484 a.a.) proteins, although the amino acid conservation is distributed across the whole length of each protein.

## Discussion

Convergent evolution is pervasive in bacterial polyamine metabolism. At one extreme are the two alternative pathways for synthesizing spermidine and spermine/thermospermine, one dependent on decarboxylated *S*-adenosylmethionine, and the other on aspartate β-semialdehyde for chain elongation [42,43]. These two pathways employ non-analogous, nonhomologous enzymes that use different chemistry. At the other extreme are PLP-dependent decarboxylases that evolved the same substrate specificity, twice, independently, from the same closely homologous protein fold, a case of pseudoconvergent evolution [11]. Evolution of the alternative form of *N*-carbamoylputrescine amidohydrolase that we have identified is more similar to the convergent evolution of ADC. In the case of ADC, proteins from five different folds perform the same chemical reaction, with the same substrate and products, using different cofactors.

The AguA–AguY pathway, which converts agmatine to putrescine via *N*-carbamoylputrescine, is different from the canonical pathway represented by AguA-NCPAH/AguB in that it is rarely found in bacteria that synthesize triamines such as spermidine. It is more prevalent in bacteria that biosynthetically accumulate putrescine from L-arginine, or those that may catabolize exogenously derived agmatine to putrescine. The AguA–AguY pathway is also considerably more phylogenetically limited than the canonical AguA–AguB pathway, being found primarily in the Actinomycetota phylum and the α-, β-, and γ-Proteobacteria classes. Its patchy distribution within the α-, β-, and γ-Proteobacteria classes and Actinomycetota phylum suggests that this pathway is not ancestral and has been acquired by horizontal gene transfer. Some *Shewanella* species contain gene clusters encoding ADC and agmatinase, indicating that these gene clusters are responsible for putrescine biosynthesis. The same species also encode gene clusters encoding either the canonical AguA–AguB or AguA–AguY pathways, sometimes including a polyamine substrate binding protein for an ABC uptake transporter. This would suggest that the AguA–AguB and AguA–AguY encoding genes are responsible for catabolism of exogenously acquired agmatine. Although it is not clear what selective pressure may have driven the evolution and spread of the AguY, a requirement for L-arginine or agmatine catabolism may have contributed.

In *P. aeruginosa*, the gene encoding the Type VI secretion system toxin Tse8, which exhibits high amino acid sequence identity to the AguY proteins, is not found in a cluster with an *aguA* gene. Instead, it is found adjacent to a gene encoding the small, toxin immunity protein Tsi8 [40]. In another part of the chromosome, *P. aeruginosa* encodes the canonical AguA and AguB proteins. It is thought that Tse8 inhibits global translation in target bacterial cells by binding to proteins in the transamidosome, an evolutionarily ancient RNA/multiprotein complex found throughout bacteria and archaea [44]. The transamidosome is required to convert glutamate-charged tRNA to glutamine-charged tRNA (Gln-tRNA). Once transferred into target bacteria through the Type VI secretion system, Tse8 binds to the GatCAB heterotrimeric enzyme complex in the transamidosome, thereby disrupting Gln-tRNA formation and inhibiting global translation. Although there is only low sequence similarity, the 3D protein structure of Tse8 is very similar to that of GatA within the GatCAB complex in the transamidosome [41]. GatA is an amidase/glutaminase that converts glutamine to glutamate and ammonia [45]. Tse8 does not exhibit GatA amidase activity [40]; however, it binds the GatCAB complex and may disrupt GatCAB function due to the similarity of the structure of Tse8 and GatA, resulting in global inhibition of protein synthesis [40]. Tse8 has likely evolved from AguY, so an intriguing possibility is that AguY evolved, directly or indirectly, from GatA. The 1-carboxybiuret amidohydrolase AtzE, which converts 1-carboxybiuret to 1,3-dicarboxyurea and ammonia, has been proposed to have evolved from GatA due to similarity of structure [46]. It is formally possible that diverse neofunctionalized descendants emerged from the ancient amidase GatA, including AguY and the Type VI secretion system toxin Tse8.

## Conclusions

Our current study has functionally identified a class of convergently evolved, alternative noncanonical *N*-carbamoylputrescine amidohydrolases in bacteria. This is a further example of the pervasive role of convergent evolution in polyamine metabolism and indicates the selective pressure to evolve new routes for polyamine biosynthesis and catabolism. The discovery that the Type VI secretion system toxin Tse8 evolved from AguY reveals the potential for functional plasticity of ancient and robust protein folds exemplified by the GatA glutamine amidohydrolase.

## Materials and methods

### Materials

*N*-carbamoylputrescine was purchased from AKos GmbH (cat. no. AKOS005067098), and putrescine (1,4-diaminobutane) dihydrochloride from Sigma-Aldrich (P7505). Plasmids for expression in *E. coli*, pETDuet-1 and pACYCDuet-1, were obtained from Novagen. Synthetic genes with *E. coli*-optimized codons were purchased from GenScript. The synthetic genes encoded the following proteins: *P. aeruginosa* AguA (GenBank protein accession no. WKE26465; 368 a.a.), *E. faecalis* AguA (EPI20829; 365 a.a.), *P. aeruginosa* NCPAH/AguB (NP_248984; 292 a.a.), *S. oneidensis* AguY (WP_011071177; 568 a.a.), *M. gilva* AguY (WP_130505540; 584 a.a.). Synthetic genes were ligated into pETDuet-1 or pACYCDuet-1 with 5′-Nde1 and 3′-Xho1 sites, expressed from a phage T7 promoter, and selected with ampicillin or chloramphenicol, respectively.

### *E. coli* strains**, growth, and gene expression conditions**

The construction of the agmatinase (*speB*) gene deletion of *E. coli* BL21 (DE3) was described previously [43]. All strains were grown twice in 2 ml of liquid, chemically defined, polyamine-free M9 minimal medium, at 37°C overnight. A 1.0-ml aliquot of culture was then centrifuged, the supernatant was discarded, and cells were resuspended in 10 ml M9 medium and grown at 37°C to OD_600nm_ = 0.5. Gene expression from pETDuet-1 and pACYCDuet-1 was induced by addition of 0.2 mM isopropyl-β-d-thiogalactopyranoside, and cultures were maintained at 16°C, overnight. Cells were then pelleted by centrifugation, and polyamines were extracted.

### Polyamine extraction from *E. coli* strains

Cultures of *E. coli* BL21*speB* expressing different plasmids were centrifuged, and pellets were washed three times by resuspension in PBS. Repelleted cells were resuspended in 200 µl of lysis buffer (100 mM MOPS pH 8.0, 50 mM NaCl, 20 mM MgCl_2_), frozen in liquid nitrogen, thawed at 37°C, and this was repeated three times. To this was added 60 µl of 40% trichloroacetic acid and, after thorough mixing, kept on ice for 5 min. Cellular debris was pelleted by centrifugation at 4°C, and the supernatant was transferred to a new tube.

### *In vitro* enzyme reactions for putrescine product detection by LC-MS/MS

Enzyme reactions with different amounts of enzyme were performed in 500 µl volumes containing 50 mM MES-NaOH (pH 7.0) buffer, 1 mM DTT, 1 mM *N*-carbamoylputrescine, and 10, 25, or 50 nM enzyme at 40°C for 60 min. Negative controls consisted of no added enzyme or 10 nM enzyme boiled for 10 min. A 200 µl aliquot of the reaction was removed, and to this was added 60 µl 40% trichloroacetic acid to stop the reaction. After centrifugation of the stopped reaction aliquot, the supernatant was diluted 1000-fold, and these samples were then used for analysis by LC-MS/MS.

### *In vitro* enzyme reactions for ammonia product detection

To detect ammonia release from enzymatic hydrolysis of *N*-carbamoylputrescine, we used the indophenol blue method for NCPAH activity described previously [47]. Each triplicate reaction contained 50 mM MES-NaOH (pH 7.0) buffer, 1 mM DTT, 0.0–2.5 mM *N*-carbamoylputrescine, and 0–30 nM enzyme at 40°C for 30 min. A 100 µl reaction aliquot was removed, and to this was added 100 µl indophenol blue reagent, which is 0.33 M sodium phenolate (prepared fresh daily), 0.01% (w/v) sodium nitroprusside (prepared fresh daily). To this was then added 1 µl 10% sodium hypochlorite, followed by immediate vigorous mixing. The mix was then heated to 100 °C for 5 min and kept on ice for 5 min. After transfer to a 96-well plate, absorbance was measured at OD_640nm_ using a Biotek Cytation 5 plate reader. A range of 0–40 µg/ml NH_4_Cl was used to develop a standard calibration curve using the same buffer and reagent. The *N*-carbamoylputrescine substrate manifests background absorbance at OD_640nm_, therefore the background signal was removed from the reaction absorbance data at the corresponding substrate concentration. The pure NH_4_Cl standard curve was used to calculate the OD_640nm_ absorbance based on NH_4_Cl concentration, which was then divided by reaction time to obtain the reaction velocity. GraphPad Prism was employed to obtain Michaelis–Menten model graph fitting, and by inputting the substrate concentration and reaction speed, the *K*_m_ and V_max_ were obtained, and then enzyme concentration was included to calculate *k*_cat_.

### LC-MS/MS quantification of underivatized *N*-carbamoylputrescine and putrescine

Pure samples of *N*-carbamoylputrescine and putrescine were used to validate identity and retention times. High-performance liquid chromatography (HPLC) was performed under reversed-phase condition using an ACE 3 C18-PFP 150×4.6 mm HPLC column, Mac-Mod, U.S.A. Column temperature, sample injection volume, and flow rate were set to 30°C, 5 μl, and 0.5 ml/min, respectively. HPLC conditions were as follows: Solvent A: water with 0.1% formic acid (v/v), LC/MS grade and Solvent B: acetonitrile with 0.1% formic acid (v/v), LC/MS grade. The gradient condition was 0–2 min: 2% B, 5–16 min: 90% B, 17 min 2% B, 30 min: 2% B. Total run time: 30 min.

LC-MS/MS mass spectrometric analyses were performed on a Sciex QTRAP 6500 + mass spectrometer equipped with an Electrospray Ionization (ESI) ion spray source. The ESI source was used in the positive mode, and ESI source conditions were set as follows: Ion Source Gas 1 (Gas 1), 40 p.s.i.; Ion Source Gas 2 (Gas 2), 35 p.s.i.; curtain gas (CUR), 45 p.s.i.; source temperature, 550°C; and ion spray voltage + 4800 V(+). The mass spectrometer was coupled to a Shimadzu HPLC (Nexera X2 LC-30AD), and the system was controlled by Analyst 1.7.3 software. Data were processed by SCIEX MultiQuant 3.0.3 software.

### Phylogenetic analysis

Homologs of the AguY and other relevant proteins were identified in bacterial proteomes using BLASTP analysis, employing the *S. oneidensis* AguY amino acid sequence (WP_011071177). Sequence alignment was performed with MAFFT [48], a Maximum Likelihood (ML) phylogenetic tree was created from the alignment using IQ-TREE [49], employing the automatic best-fit amino acid substitution model and with 1000 ultrafast bootstraps analysis [50]. The ML phylogenetic tree was visualized with iTOL [51].

## Data Availability

All relevant data are included in the main text.

## Abbreviations

ADC: arginine decarboxylase
AguA: agmatine deiminase
AguB: canonical *N*-carbamoylputrescine amidohydrolase
AguY: alternative noncanonical *N*-carbamoylputrescine amidohydrolase
GatA: L-glutamine amidohydrolase
Gln-tRNA: glutamine-charged tRNA
HPLC: high-performance liquid chromatography
ML: maximum likelihood
NCPAH: *N*-carbamoylputrescine amidohydrolase
ORF: open reading frame
PLP: pyridoxal 5′-phosphate
SpeB: agmatinase/agmatine ureohydrolase
